# Acknowledgment to Reviewers of *Medical Sciences* in 2021

**DOI:** 10.3390/medsci10010008

**Published:** 2022-01-27

**Authors:** Medical Sciences Editorial Office

**Affiliations:** MDPI AG, St. Alban-Anlage 66, 4052 Basel, Switzerland

Rigorous peer-reviews are the basis of high-quality academic publishing. Thanks to the great efforts of our reviewers, *Medical Sciences* was able to maintain its standards for the high quality of its published papers. Thanks to the contribution of our reviewers, in 2021, the median time to first decision was 24 days and the median time to publication was 54 days. The editors would like to extend their gratitude and recognition to the following reviewers for their precious time and dedication, regardless of whether the papers they reviewed were finally published:
Agresta, FerdinandoLi, MingqingAlbendín-García, LuisLin, Han-JiaAlbertini, MariaLlorente, Carlos MartinAl-Rihani, Sweilem B.López-Muñoz, EuniceAndrej, JanezLosi, PaolaAndres, AntonioLui, FaustaAntonakakis, MariosManzo, CiroAstl, JaromírMars, MauriceBaldassarre, Maria P. A.Masqué-Soler, NeusBar-Haim, ErezMatkowski, AdamBattistelli, MichelaMazzanti, MicheleBeninati, SimoneMeinhardt, MatthiasBentel, Jacqueline M.Meng, YanBertelloni, FabrizioMiller, Douglas C.Boccia, GiovanniMongirdienė, AušraBolanowski, MarekMorselli, SimoneBowsher, GemmaMytych, JenniferBraicu, CorneliaNakanishi, NobutoBrieger, AngelaNakazawa, TsutomuBrown, JustinNapoli, ChristianBukhary, Dalea M.Nepogodiev, DmitriCalabrò, Rocco SalvatoreNiku, MikaelCarames, João Manuel MendesNiland, StephanCarbone, AntoninoNowak, BeataCascella, MarcoOtsuki, TakemiCervelli, ManuelaOzmen, AsliChen, PingPallag, AnnamáriaChin, Kok YongPalmieri, ErikaCho, Hea-YoungPandey, SadanandChrist, LisaParmar, MayurColavito, Sierra A.Pasek, JarosławCostantini, LaraPasquali, ClaudioDeigner, Hans-PeterPata, FrancescoDelgado-Losada, María LuisaPerdomo, GermanDermitzakis, Emmanouil V.Pereira, Carlos A. B.Devesa, JesúsPfeiffer, PerDo, Sun HeePisla, AdrianDoddamani, RajivPrázný, MartinDunkley, Benjamin T.Psaroulaki, AnnaEdmunds, Lia R.Ramprasath, TharmarajanElemary, MohamedRappelli, GiorgioEmami, MehrdadRather, IrfanEna, JavierRaval, YashErnsberger, PaulRazo, RodrigoEscola-Gil, Joan CarlesRivadeneyra-Domínguez, EduardoExbrayat, Jean-MarieRoda, ElisaFawzy, ManalRomanelli, AntonioFeier, HoreaRomanini, AntonellaFeith, DavidRoque, FátimaFernández-Barat, LaiaSahgal, PranshuFierro, Maria TeresaSammarco, GiuseppeFischer, FlorianSchoenberg, FredericFortofoiu, Mircea-CatalinSebastiani, GiovanniFranco, Roberto S.Sevcikova, SabinaGairhe, SalinaShah, ShamikGarcía-Silva, SusanaShen, Che-HungGatto, RodolfoShiao, Chih-ChungGica, NicolaeShiraki, MasatakaGiménez-Bastida, Juan AntonioSignorini, CinziaGrande, FedoraSmith, AlexandraGreenhalgh, DavidSong, JiajiaHeidor, RenatoSopena, JoaquínHeier, ChristophStjepanovic, DanielHirono, KeiichiStocker, ClaireHofer, Sebastian J.Storici, PaolaHongo, MichioStratmann, BerndHoráková, DagmarSzultka-Młyńska, MałgorzataHsu, Tsung-ITak, Erwin C. P. M.Hu, MinghanTanoubi, IssamHuang, I.-S. WadeTardáguila-García, AroaHuang, Weei-YuarnTender, Gabriel ClaudiuIngrid, TonhajzerováTerčelj, MarjetaJankovic, MilicaTinelli, AndreaJanowska, AgataTinnirello, AndreaJörns, AnneTosti, GiulioJović, DanicaTrujillo Silva, Daniela JoyceKang, Hee EunTsai, Han-MouKarousi, ParaskeviTuomilehto, JaakoKasprzak, AldonaTurriziani, OmbrettaKawanami, DaijiVassiliou, Alice GeorgiaKim, Ryong NamVelasco, MaríaKim, Sang GuenVerhoeven, AdrieKlein, Mark A.Waasdorp, MaaikeKniotek, MonikaWan, ShaohuaKobeissy, FirasWang, ShenqiKojima-Yuasa, AkikoWiedlocha, AntoniLaguna, Juan C.Woźniak-Budych, MartaLapolla, AnnunziataXiang, KuanhuiLe Tissier, Paul R.Xu, JianLewandowski, Łukasz B.Zandonadi, Renata


